# A Faith-Based Intervention to Reduce Blood Pressure in Underserved Metropolitan New York Immigrant Communities

**DOI:** 10.5888/pcd16.180618

**Published:** 2019-08-08

**Authors:** Stella S. Yi, Laura C. Wyatt, Shilpa Patel, Catherine Choy, Ritu Dhar, Jennifer M. Zanowiak, Harmanpreet Chuhan, M.D. Taher, Maryjoy Garcia, Rucha Kavathe, Sara Kim, Simona C. Kwon, Nadia S. Islam

**Affiliations:** 1 Department of Population Health, New York University School of Medicine, New York, New York; 2 California State University, Fullerton, California; 3 Kalusugan Coalition, Woodside, New York; 4 United Sikhs, New York, New York; 5 Korean Community Services of Metropolitan New York, Inc, New York, New York

## Abstract

Minority populations, including Asian Americans, face disparities in hypertension compared with non-Hispanic whites. This underscores the need for culturally adapted programs in settings that reach Asian American communities, such as faith-based organizations. We worked collaboratively with community partners to culturally adapt and implement an evidence-based community blood pressure monitoring program for Asian Americans (Asian Indians, Koreans, Filipinos, and Bangladeshis) in metropolitan New York during 2015 and 2016. The program included regularly scheduled volunteer-led screening and counseling events with congregants at faith-based organizations. Among participants with complete 6-month data (n = 348), health-related self-efficacy significantly improved after 6 months, and systolic and diastolic blood pressure was significantly reduced in some subgroups; reductions were highest in participants who self-reported a previous diagnosis of hypertension. Among Asian Americans, faith-based programs may be a replicable, low-cost, sustainable way to increase health-related self-efficacy and decrease blood pressure, specifically among individuals with self-reported hypertension.

**Summary**

**What is already known on this topic?**

Minority populations, including Asian Americans, face disparities in hypertension compared with non-Hispanic whites, underscoring the need for culturally adapted programs in settings that reach Asian American communities.

**What is added by this report?**

We collaborated with our community partners to culturally adapt and implement an evidence-based community blood pressure monitoring program for Asian Americans (Asian Indian, Korean, Filipino, Bangladeshi) in metropolitan New York and New Jersey in 2015 and 2016 across 12 faith-based organizations.

**What are the implications for public health practice?**

Faith-based programs targeting Asian Americans may be a replicable, low-cost, sustainable way to increase health-related self-efficacy and for decreasing blood pressure among Asian Americans with diagnosed hypertension.

**MEDSCAPE CME**

In support of improving patient care, this activity has been planned and implemented by Medscape, LLC and *Preventing Chronic Disease*. Medscape, LLC is jointly accredited by the Accreditation Council for Continuing Medical Education (ACCME), the Accreditation Council for Pharmacy Education (ACPE), and the American Nurses Credentialing Center (ANCC), to provide continuing education for the healthcare team.

Medscape, LLC designates this Journal-based CME activity for a maximum of 1.00 AMA PRA Category 1 Credit(s)™. Physicians should claim only the credit commensurate with the extent of their participation in the activity.

Successful completion of this CME activity, which includes participation in the evaluation component, enables the participant to earn up to 1.0 MOC points in the American Board of Internal Medicine’s (ABIM) Maintenance of Certification (MOC) program. Participants will earn MOC points equivalent to the amount of CME credits claimed for the activity. It is the CME activity provider’s responsibility to submit participant completion information to ACCME for the purpose of granting ABIM MOC credit.

**Release date: August 8, 2019; Expiration date: August 8, 2020**

**Learning Objectives**

Upon completion of this activity, participants will be able to:

1. Distinguish elements of the current community-based intervention
2. Assess the effects of the current intervention on blood pressure variables
3. Analyze particular groups who benefited from the current community-based intervention
4. Evaluate the current intervention among individuals with elevated blood pressure at baseline

**EDITOR**

Camille Martin

Editor, *Preventing Chronic Disease*

Disclosure: Camille Martin has disclosed no relevant financial relationships.

**CME AUTHOR**

Charles P. Vega, MD

Health Sciences Clinical Professor of Family Medicine

University of California, Irvine School of Medicine

Irvine, California

Disclosure: Charles P. Vega, MD, has disclosed the following relevant financial relationships:

Served as an advisor or consultant for: Johnson & Johnson Pharmaceutical Research & Development, L.L.C.; Genentech; GlaxoSmithKline

Served as a speaker or a member of a speakers bureau for: Shire

**AUTHORS**

Stella S. Yi, PhD

Department of Population Health, NYU School of Medicine, New York, NY

Disclosure: Stella S. Yi, PhD, has disclosed no relevant financial relationships.

Laura C. Wyatt, MPH

Department of Population Health, NYU School of Medicine, New York, NY

Disclosure: Laura C. Wyatt, MPH, has disclosed no relevant financial relationships.

Shilpa Patel, PhD

Department of Population Health, NYU School of Medicine, New York, NY

Disclosure: Shilpa Patel, PhD, has disclosed no relevant financial relationships.

Catherine Choy, MPH^†^

Department of Population Health, NYU School of Medicine, New York, NY

Disclosure: Catherine Choy, MPH, has disclosed no relevant financial relationships.

†Deceased.

Ritu Dhar, BS

Department of Population Health, NYU School of Medicine, New York, NY

Disclosure: Ritu Dhar, BS, has disclosed no relevant financial relationships.

Jennifer M. Zanowiak, MA

Department of Population Health, NYU School of Medicine, New York, NY

Disclosure: Jennifer M. Zanowiak, MA, has disclosed no relevant financial relationships.

Harmanpreet Chauhan, MPH

California State University, Fullerton, CA

Disclosure: Harmanpreet Chauhan, MPH, has disclosed no relevant financial relationships.

M.D. Taher, MPH

Department of Population Health, NYU School of Medicine, New York, NY

Disclosure: M.D. Taher, MPH, has disclosed no relevant financial relationships.

Maryjoy Garcia, DNP, RN

Kalusugan Coalition Woodside, Queens, NY

Disclosure: Maryjoy Garcia, DNP, RN, has disclosed no relevant financial relationships.

Rucha Kavathe, PhD

UNITED SIKHS, New York, NY

Disclosure: Rucha Kavathe, PhD, has disclosed no relevant financial relationships.

Sara Kim, MPH

Korean Community Services of Metropolitan New York, Inc., New York, NY

Disclosure: Sara Kim, MPH, has disclosed no relevant financial relationships.

Simona C. Kwon, DrPH

Department of Population Health, NYU School of Medicine, New York, NY

Disclosure: Simona C. Kwon, DrPH, has disclosed no relevant financial relationships.

Nadia S. Islam, PhD

Department of Population Health, NYU School of Medicine, New York, NY

Disclosure: Nadia S. Islam, PhD, has disclosed no relevant financial relationships.

## Introduction

Nearly one-third of the US adult population has hypertension (1). Minority populations, including African Americans, Latinos, and Asian Americans, face disparities in hypertension and related cardiovascular disease (CVD) outcomes compared with non-Hispanic whites (1–3). Despite these findings, there is limited research on interventions to address high blood pressure in community settings among minority groups such as Asian Americans. Furthermore, research suggests that declines in CVD mortality rates observed in the broader US population in the past decade are not reflected for Asian Americans, indicating that current activities may not be reaching these groups (4,5).

Keep on Track (KOT), a program developed by the New York City Department of Health and Mental Hygiene (NYC DOHMH), is a volunteer-run, community-based blood pressure monitoring program that aims to lower blood pressure in community-dwelling adults. KOT was first successfully implemented and evaluated through senior center programming in New York City. The involvement of peer volunteers was central to the program (6).

Faith-based organizations (FBOs) may be effective settings for implementing health promotion and prevention strategies in Asian American communities. A 2012 Pew Research Center survey found that Asian Americans report a high level of FBO engagement but also report substantial differences in religious affiliation within and across subgroups (7). Research suggests that FBOs are successful in delivering health promotion programs because of their roles as trusted community centers and their ability to leverage infrastructure and social capital resources (eg, health ministries, regular congregant contact) for such programs (8,9). However, there are limited studies on faith-based health promotion interventions among Asian American communities (8,10,11).

## Purpose and Objectives

The Racial and Ethnic Approaches to Community Health For Asian Americans (REACH FAR) project, led by a coalition composed of academic, government, and community-based organizations, culturally adapted and implemented the KOT program for 4 Asian American faith-based communities in metropolitan New York City and New Jersey during 2015 and 2016 (12). We present program findings, overall and stratified by Asian American subgroup and hypertension status at baseline.

## Intervention Approach

The REACH FAR KOT program approach was informed by prior work in senior centers (6) and drew from social cognitive theory, which integrates Bandura’s construct of self-efficacy and the factors that influence the confidence a person has to make a behavioral change (13). Our program began in September 2015 and was implemented in 12 FBOs in New York City and New Jersey: 3 Sikh gurdwaras, 2 Bangladeshi mosques, 3 Filipino churches, 3 Korean churches, and one Bangladeshi FBO-co-located senior center. Sites were selected on the basis of pre-existing relationships with coalition partners. During the engagement stage of the implementation process, community partners met with FBO leadership and key congregation members to introduce the program and develop an implementation plan aligned with each FBO’s organizational structure. Recruited FBOs received a site honorarium for participation and signed a memorandum of understanding agreement that outlined program and evaluation activities. Next, NYC DOHMH–trained bilingual consultants recruited a team of FBO volunteers (ie, congregation members already active in existing health or wellness committees) to implement and deliver the program at each site. Recruited volunteers (96 individuals across the 12 sites) participated in a 2-day NYC DOHMH train-the-trainer program, which included topics on understanding the health effects of hypertension, program logistics, and operational duties.

Volunteers advertised the REACH FAR KOT program launch through FBO communication channels, including fliers, newsletters, listservs, and congregation leadership announcements. At each launch event, volunteers conducted a free blood pressure screening for all interested congregation members aged 18 to 85 years and recorded blood pressure measurements on a tracking card. After the screening, volunteers provided in-language, culturally tailored lifestyle counseling on maintaining healthy blood pressure and weight management, which included reviewing healthy eating tips and examples of healthy plates using common cultural foods (14). KOT volunteers also provided health coaching to participants to enhance their confidence in overcoming access barriers and their ability to ask and understand information about hypertension during clinical encounters. Participants were provided with translated blood pressure “passports” that documented measurements, medications, and questions that participants were encouraged to share with providers. Congregation members who had a blood pressure reading of 140/90 mm Hg or higher were advised to follow up with their primary care physician or were provided a referral to care. Post-launch, volunteers held regular screening events open to the entire congregation; returning congregants’ blood pressure measurements were tracked on the same screening record card kept at the FBO site. The frequency of regularly held screening events post-launch depended on the FBO site’s preference and capacity. For example, 1 site held KOT events every 2 weeks, while other sites held events quarterly.

All KOT program participants were invited to enroll in the KOT program evaluation through recruitment events held before or after regular FBO services. Pregnant women were not eligible to participate. People who consented to participate completed surveys at baseline and 6 months post-launch administered by program evaluation staff and received a gift card incentive. All activities were approved by the New York University School of Medicine institutional review board.

## Evaluation Methods

Outcomes, assessed at baseline and 6-month follow-up, were 1) systolic and diastolic blood pressure (SBP and DBP), 2) a blood pressure screening in the last 6 months, 3) a doctor’s visit in the last 6 months, and 4) health-related self-efficacy.

Blood pressure measurements were taken by trained volunteers at the initial KOT event and each subsequent screening event by using the Omron Model BP785 (Omron Corporation). Because multiple blood pressure readings were not consistently taken, the first blood pressure measurements were used for all analyses.

Baseline and 6-month surveys assessed blood pressure outcomes (blood pressure screening history, diagnosis of hypertension, and blood pressure medication use); demographics (age, sex, nativity, ethnicity); physiologic measures (self-reported height and weight); and health-related self-efficacy. A health-related self-efficacy scale was adapted from Bandura to measure health-related decision-making behaviors (13). Eight questions specific to health-related self-efficacy were confidence in 1) making decisions regarding health, 2) asking about health issues, 3) going to the doctor alone, 4) picking up the telephone to find out where to go for care, 5) knowing where to get medical attention, 6) needing others to accompany you, 7) finding your way around the city on public transportation, and 8) having the right to use family income to take care of medical needs. Scores ranged from 1 to 4, and 4 represented highest self-efficacy. We used alternative World Health Organization-recommended cut-off points to assess body mass index (15).

Differences in means (SBP, DBP, health-related self-efficacy) were assessed using *t* tests; differences in proportions (blood pressure screening or doctor’s visit within 6 months) were assessed using χ^2^ tests. Analyses were conducted for all participants and stratified by Asian American subgroups as well as self-reported hypertension at baseline. Self-reported hypertension was determined by asking the question, “Has a doctor or other health professional ever told you that you have high blood pressure?” We also stratified by elevated blood pressure at baseline (SBP ≥160 mm Hg and/or DBP ≥100 mm Hg) as a conservative proxy for including those with uncontrolled hypertension. Changes in SBP and DBP were assessed longitudinally by examining the slope of blood pressure change over time across multiple readings and using generalized estimating equations among all participants (with readings at points in addition to baseline and follow-up). Finally, we descriptively analyzed program data to determine program feasibility, including average number of screening events and number of people screened per event.

## Results

A total of 1,653 congregants were screened across all sites and screening events. A subset of participants were enrolled, consented, and completed a baseline survey (n = 719). A total of 348 participants (48.4%) completed follow-up surveys (Asian Indian = 66, Bangladeshi = 52, Filipino = 47, Korean = 168, Other/Not reported = 15). Participants were predominantly aged 55 years or more, female, and foreign-born (Table 1). Obesity was higher among South Asian Americans (Asian Indians and Bangladeshis) than among Filipino and Korean American congregants. Self-reported hypertension and having a regular place of care varied across the Asian American subgroups.

**Table 1:** Demographic Characteristics of Participants With Baseline and Follow-Up Data, REACH FAR Keep on Track, New York City/New Jersey, 2015–2016^a^

Characteristic	All (N = 348)	Asian Indian (n = 66)	Bangladeshi (n = 52)	Filipino (n = 47)	Korean (n = 168)	*P* Value^b^
	No. (%)					
**Age, y**						<.001
18–34	23 (6.6)	5 (7.6)	6 (11.5)	8 (17.0)	3 (1.8)	
35–44	26 (7.5)	10 (15.2)	4 (7.7)	8 (17.0)	4 (2.4)	
45–54	66 (19.0)	17 (25.8)	9 (17.3)	13 (27.7)	26 (15.5)	
55–64	94 (27.0)	19 (28.8)	21 (40.4)	14 (29.8)	34 (20.2)	
≥65	121 (34.8)	11 (16.7)	11 (21.2)	4 (8.5)	91 (54.2)	
Missing	18 (5.2)	4 (6.1)	1 (1.9)	0	10 (6.0)	
**Sex**						
Male	124 (35.8)	18 (27.7)	27 (51.9)	16 (34.0)	56 (33.3)	.04
Female	222 (64.2)	47 (72.3)	25 (48.1)	31 (66.0)	112 (66.7)	
**Nativity**						
Foreign-born	334 (96.5)	64 (97.0)	52 (100.0)	44 (93.6)	168 (100.0)	.006
US-born	12 (3.5)	2 (3.0)	0	3 (6.4)	0	
**Baseline BMI^c^**						
Underweight or normal weight^d^	109 (32.5)	10 (15.4)	8 (17.4)	17 (36.2)	71 (43.0)	<.001
Overweight	149 (44.5)	29 (44.6)	21 (45.7)	23 (46.8)	71 (43.0)	
Obese	77 (23.0)	26 (40.0)	17 (37.0)	8 (17.0)	23 (13.9)	
**Blood pressure screening in last 6 months**	257 (74.5)	54 (85.7)	46 (90.2)	30 (68.2)	115 (69.7)	.003
**Doctor’s visit in last 6 months**	236 (68.0)	53 (81.5)	48 (92.3)	22 (46.8)	104 (63.0)	<.001
**Ever been told had high blood pressure**	146 (42.2)	20 (30.3)	32 (61.5)	19 (40.4)	68 (41.0)	.007
**Has regular place of care**	256 (81.0)	57 (96.6)	44 (91.7)	33 (76.7)	113 (72.9)	<.001

Abbreviations: BMI, body mass index; REACH FAR, Racial and Ethnic Approaches to Community Health for Asian Americans.

a Total sample for Asian subgroups does not sum to 348 because 15 participants reported they were another ethnicity or did not report ethnicity.

b χ^2^ tests across the 4 Asian subgroups; does not include missing values.

c Classified based on World Health Organization BMI (kg/m^2^) alternative cut-offs for Asian populations: underweight/normal weight, <23; overweight, 23.0 to <27.5; obese, ≥27.5. When using standard BMI definition: underweight/normal weight, <25.0 (53.4%); overweight, 25.0 to <30 (33.6%); obese, ≥30.0 (13.0%); 11.5% of Bangladeshi BMI measurements were missing.

d Four participants were classified as underweight (<18.5 kg/m^2^).

On average, 10.5% of congregants participated across the 12 sites, varying from 4.6% of congregants at Bangladeshi sites to 54.7% of congregants at Filipino sites. Sites held an average of 9 screenings across the 6-month period (range, 2–12). Across sites, an average of 25 congregants were screened per event, and each screening event lasted 4 hours. Since the launch, the KOT program has been maintained at 11 of 12 sites. When comparing individuals who completed 6-month follow-up with those who did not complete follow-up, Korean Americans were significantly more likely to complete than other subgroups, as were women (overall and Asian Indian) and older participants (overall and Korean).

Between baseline and 6 months, mean SBP decreased significantly (128.4 mm Hg to 126.7 mm Hg, *P* = .03); blood pressure screening increased significantly (74.5% to 81.2%, *P* = .03), as did mean health-related self-efficacy (3.1 to 3.3, *P* < .001) (Table 2). Results varied by Asian American subgroup. Large, significant improvements were observed among Bangladeshi American participants for mean SBP (125.8 mm Hg to 119.5 mm Hg, *P* = .007) and mean DBP (79.0 mm Hg to 75.2 mm Hg, *P* = .004). Filipino American participants had a significant increase in doctor’s visits in the last 6 months (46.8% to 67.4%, *P* = .04), and health-related self-efficacy significantly improved among Korean American participants (3.0 to 3.2, *P* = .004).

**Table 2:** Baseline and Follow-Up BP Variables and Health-Related Self-Efficacy of Participants (N = 348), REACH FAR Keep on Track, New York City/New Jersey, 2015–2016^a^

Variable	Baseline	Follow-Up	*P* Value
**All (N = 348)**			
BP screening in last 6 months	257 (74.5)	281 (81.2)	.03
Doctor's visit in last 6 months	236 (68.0)	247 (71.2)	.36
Health-related self-efficacy, mean (SD)	3.1 (0.6)	3.3 (0.5)	<.001
SBP, mm Hg, mean (SD)	128.4 (18.2)	126.7 (17.2)	.03
DBP, mm Hg, mean (SD)	79.7 (10.6)	78.7 (9.4)	.05
**Asian Indian (n = 66)**			
BP screening in last 6 months	54 (84.4)	60 (90.9)	.26
Doctor's visit in last 6 months	53 (81.5)	58 (87.9)	.31
Health-related self-efficacy, mean (SD)	3.2 (0.5)	3.3 (0.6)	.20
SBP, mm Hg, mean (SD)	126.3 (16.9)	126.4 (15.4)	.95
DBP, mm Hg, mean (SD)	81.8 (10.1)	81.2 (8.7)	.51
**Bangladeshi (n = 52)**			
BP screening in last 6 months	46 (90.2)	45 (88.2)	.75
Doctor's visit in last 6 months	48 (92.3)	46 (88.5)	.51
Health-related self-efficacy, mean (SD)	3.1 (0.6)	3.3 (0.5)	.06
SBP, mm Hg, mean (SD)	125.8 (20.5)	119.5 (16.0)	.007
DBP, mm Hg, mean (SD)	79.0 (12.4)	75.2 (9.5)	.004
**Filipino (n = 47)**			
BP screening in last 6 months	115 (68.5)	39 (83.0)	.04
Doctor's visit in last 6 months	22 (46.8)	31 (67.4)	.04
Health-related self-efficacy, mean (SD)	3.4 (0.5)	3.4 (0.3)	.99
SBP, mm Hg, mean (SD)	118.7 (14.6)	116.0 (12.5)	.17
DBP, mm Hg, mean (SD)	77.4 (9.3)	77.2 (8.4)	.88
**Korean (n = 168)**			
BP screening in last 6 months	115 (68.5)	128 (76.6)	.09
Doctor's visit in last 6 months	104 (61.9)	103 (61.3)	.75
Health-related self-efficacy, mean (SD)	3.0 (0.5)	3.2 (0.4)	.004
SBP, mm Hg, mean (SD)	132.3 (17.9)	131.8 (17.7)	.66
DBP, mm Hg, mean (SD)	79.7 (10.5)	79.3 (9.4)	.64
**Self-reported diagnosis of hypertension at baseline (n = 146)**			
BP screening in last 6 months	125 (86.8)	131 (90.3)	.34
Doctor's visit in last 6 months	118 (80.8)	121 (83.4)	.56
Health-related self-efficacy, mean (SD)	3.1 (0.6)	3.2 (0.5)	.04
SBP, mm Hg, mean (SD)	135.3 (18.1)	131.4 (16.8)	.005
DBP, mm Hg, mean (SD)	81.9 (11.1)	79.5 (9.9)	.01
**Self-reported diagnosis of no hypertension at baseline (n = 200)**			
BP screening in last 6 months	130 (65.3)	148 (74.4)	.049
Doctor's visit in last 6 months	116 (58.3)	124 (62.0)	.45
Health-related self-efficacy, mean (SD)	3.2 (0.5)	3.3 (0.4)	.005
SBP, mm Hg, mean (SD)	123.4 (16.5)	123.1 (16.8)	.81
DBP, mm Hg, mean (SD)	78.1 (10.0)	78.1 (9.0)	.93

Abbreviations: BP, blood pressure; DBP, diastolic blood pressure; SBP, systolic blood pressure; SD, standard deviation.

a Values expressed as number (%) unless otherwise indicated. Total sample for Asian subgroups does not total 348 because 15 participants reported they were another ethnicity or did not report ethnicity. Total sample for hypertension does not total 348 because 2 participants responded “don’t know.”

The REACH FAR KOT program appeared to be effective among participants with self-reported hypertension. Among these participants, mean SBP and DBP decreased significantly from baseline to 6 months among participants (−3.9 mm Hg, *P* = .005; −2.4 mm Hg, *P* = .01, respectively); and mean health-related self-efficacy increased significantly (3.1 to 3.2, *P* = .04). Participants without self-reported hypertension also had significant changes in health-related self-efficacy and blood pressure screening.

Finally, among 24 participants with elevated blood pressure at baseline (SBP ≥160 and/or DBP ≥100), mean SBP and DBP significantly decreased (−16.7 mm Hg, *P* < .001; −8.3 mm Hg, *P* = .02, respectively). No significant changes in blood pressure were observed over time when using longitudinal data collected from all program participants and across all screening events (results not shown).

## Implications for Public Health

Our analysis demonstrates that faith-based programs may be an effective way to increase health-related self-efficacy among Asian Americans and improve blood pressure among certain Asian American subgroups or among those with self-reported hypertension. Blood pressure significantly improved for Bangladeshis after KOT program implementation. This result may be due to the high blood pressure at baseline for this group, but it may also result from their unique sociodemographic profile in New York City. Bangladeshis have lower rates of English proficiency and income compared with other racial/ethnic subgroups in New York City, which may affect use of health care resources related to hypertension and CVD (16). Community-based programs such as KOT that provide free in-language screenings may be especially relevant and useful for Bangladeshis and groups with similar health disparities. Furthermore, these programs may help reduce SBP and DBP among those with uncontrolled blood pressure at baseline, even using a conservative elevated level (SBP/DBP, 160/100 mm Hg).

Our study has limitations. Caution should be used in interpreting the large changes in blood pressure that were observed, because regression to the mean may have occurred without a control group. The findings, however, provide a promising avenue for addressing undiagnosed elevated blood pressure, particularly for Asian American communities who may not have access to regular care and who may be more likely to be reached in trusted community settings such as FBOs. Additionally, because this evaluation used a pre–post design, our internal validity is limited.

However, our results indicate high program feasibility and sustainability, which is in large part due to the integration of sustainability planning throughout each KOT program site using a capacity-building approach. For example, training FBO volunteers who were known and trusted to deliver the blood pressure screening program built the FBO’s capacity to engage in and sustain broader health promotion activities. Given the change in recent guidelines stating that high blood pressure should be treated earlier with lifestyle changes and/or medication at blood pressure readings of 130/80 mm Hg rather than 140/90 mm Hg, at-risk populations will increasingly need to be linked to blood pressure monitoring and health promotion resources at the community level. Programs such as KOT, which provide community-based health counseling and blood pressure screening, may offer easily replicable, low-cost, sustainable programs for underserved communities.

## Post-Test Information

To obtain credit, you should first read the journal article. After reading the article, you should be able to answer the following, related, multiple-choice questions. To complete the questions (with a minimum 75% passing score) and earn continuing medical education (CME) credit, please go to http://www.medscape.org/journal/pcd. Credit cannot be obtained for tests completed on paper, although you may use the worksheet below to keep a record of your answers.

You must be a registered user on http://www.medscape.org. If you are not registered on http://www.medscape.org, please click on the “Register” link on the right hand side of the website.

Only one answer is correct for each question. Once you successfully answer all post-test questions, you will be able to view and/or print your certificate. For questions regarding this activity, contact the accredited provider, CME@medscape.net. For technical assistance, contact CME@medscape.net. American Medical Association’s Physician’s Recognition Award (AMA PRA) credits are accepted in the US as evidence of participation in CME activities. For further information on this award, please go to https://www.ama-assn.org. The AMA has determined that physicians not licensed in the US who participate in this CME activity are eligible for *AMA PRA Category 1 Credits™*. Through agreements that the AMA has made with agencies in some countries, AMA PRA credit may be acceptable as evidence of participation in CME activities. If you are not licensed in the US, please complete the questions online, print the AMA PRA CME credit certificate, and present it to your national medical association for review.

## Post-Test Questions

### Study Title: A Faith-Based Intervention to Reduce Blood Pressure in Underserved Metropolitan New York Immigrant Communities

#### CME Questions

1. You want to replicate the Keep On Track program described in the current study by Stella and colleagues. What should you consider regarding the intervention in this study?
  - It targeted senior centers
  - It was focused on African Americans
  - Participants were encouraged to share their blood pressure data with healthcare providers
  - Counseling to individual participants was provided by healthcare professionals
2. Which one of the following options was a principal outcome of the Keep On Track intervention in the current study?
  - Increase in blood pressure screening and no change in mean systolic blood pressure levels
  - Increase in blood pressure screening and mild reduction in mean systolic blood pressure levels
  - No change in blood pressure screening rates and increase in mean systolic blood pressure levels
  - No change in blood pressure screening rates or mean systolic blood pressure levels
3. Which one of the following groups experienced the biggest reduction in average systolic blood pressure in the current study by Stella and colleagues?
  - Asian Indians
  - Bangladeshi
  - Filipino
  - Korean
4. Your target population has a high baseline rate of hypertension. Which one of the following outcomes was noted in the subgroup with elevated blood pressure at baseline in the current study?
  - An increase in the rate of blood pressure screening
  - An increase in physician's office visits
  - A reduction in the average systolic blood pressure
  - No change in health-related self-efficacy
